# Data Curation can Improve the Prediction Accuracy of Metabolic Intrinsic Clearance

**DOI:** 10.1002/minf.201800086

**Published:** 2018-09-24

**Authors:** Tsuyoshi Esaki, Reiko Watanabe, Hitoshi Kawashima, Rikiya Ohashi, Yayoi Natsume‐Kitatani, Chioko Nagao, Kenji Mizuguchi

**Affiliations:** ^1^ Laboratory of Bioinformatics National Institutes of Biomedical Innovation, Health and Nutrition 7-6-8 Saito-Asagi, Ibaraki Osaka 567-0085 Japan; ^2^ Discovery Technology Laboratories Mitsubishi Tanabe Pharma Corporation 2-2-50 Kawagishi, Toda Saitama 335-8505 Japan; ^3^ Laboratory of In-silico Drug Design, Center for Drug Design Research National Institutes of Biomedical Innovation, Health and Nutrition 7-6-8 Saito-Asagi, Ibaraki Osaka 567-0058 Japan

**Keywords:** Intrinsic clearance, Drug discovery, Machine learning, Metabolic stability, Molecular modeling

## Abstract

A key consideration at the screening stages of drug discovery is in vitro metabolic stability, often measured in human liver microsomes. Computational prediction models can be built using a large quantity of experimental data available from public databases, but these databases typically contain data measured using various protocols in different laboratories, raising the issue of data quality. In this study, we retrieved the intrinsic clearance (*CL*_int_) measurements from an open database and performed extensive manual curation. Then, chemical descriptors were calculated using freely available software, and prediction models were built using machine learning algorithms. The models trained on the curated data showed better performance than those trained on the non‐curated data and achieved performance comparable to previously published models, showing the importance of manual curation in data preparation. The curated data were made available, to make our models fully reproducible.

## 1 Introduction

Various high‐throughput screening methods have been proposed to prioritize compounds in the early stages of drug discovery. In this process, in vitro metabolic stability in human liver microsomes (HLMs) is an extremely important factor to influence the potential of drugs. A key strategy employed over the last decade is early determination and prediction of metabolic stability.^1^

Previous studies on the development of *in silico* models for metabolic stability shared common features including: (1) closed datasets (typically derived from a company's in‐house data), (2) the use of commercial software to calculate the descriptors of compounds, (3) the target property being either intrinsic clearance (*CL*_int_)^2^, ^3^, ^4^ or half‐life (T_1/2_)^5^, ^6^, ^7^, ^8^ and (4) the resulting models being mostly binary classifiers (stable or unstable).^2^, ^3^,^5^, ^6^, ^7^, ^8^

While standardized in‐house protocols ensure data quality and can contribute to high performance models,^5^ these models are typically presented with little or no information about the compounds used for training. Furthermore, commercial software can be prohibitive for many academic scientists. These issues make it difficult or impossible to reproduce published studies. While T_1/2_, the time for a compound to reduce to half of its original amount, depends on experimental conditions, *CL*_int_, expressed in either mL/min/kg (body weight) or μL/min/mg (microsomal protein weight), represents a value normalized by key experimental factors and therefore, is a more suitable property for building predictive models for metabolic stability. Binary classifiers are useful for applying a quick filter but it is often desirable to retain compounds in the “moderate category” between stable and unstable, so that these compounds can be later evaluated more properly and modified to improve their metabolic stability.

To address these issues, we have built three‐class classifiers for *CL*_int_, trained on data collected from a public source and using freely available software only. A major challenge was to ensure quality and quantity for data from public sources; a large number of *CL*_int_ measurements are available from public databases but they were obtained in varying experimental conditions including: microsomal protein concentration, incubation time, time points, buffer composition, quality control, and the units of experimental data.^1^ Our solution was to perform extensive manual curation and collect data that satisfied a selected set of criteria. We demonstrate that the use of the curated data enabled us to build models with higher performance than those trained on a non‐curated dataset.

## 2 Material and Methods

### 2.1 Collection of Experimental data

**Scheme 1:**
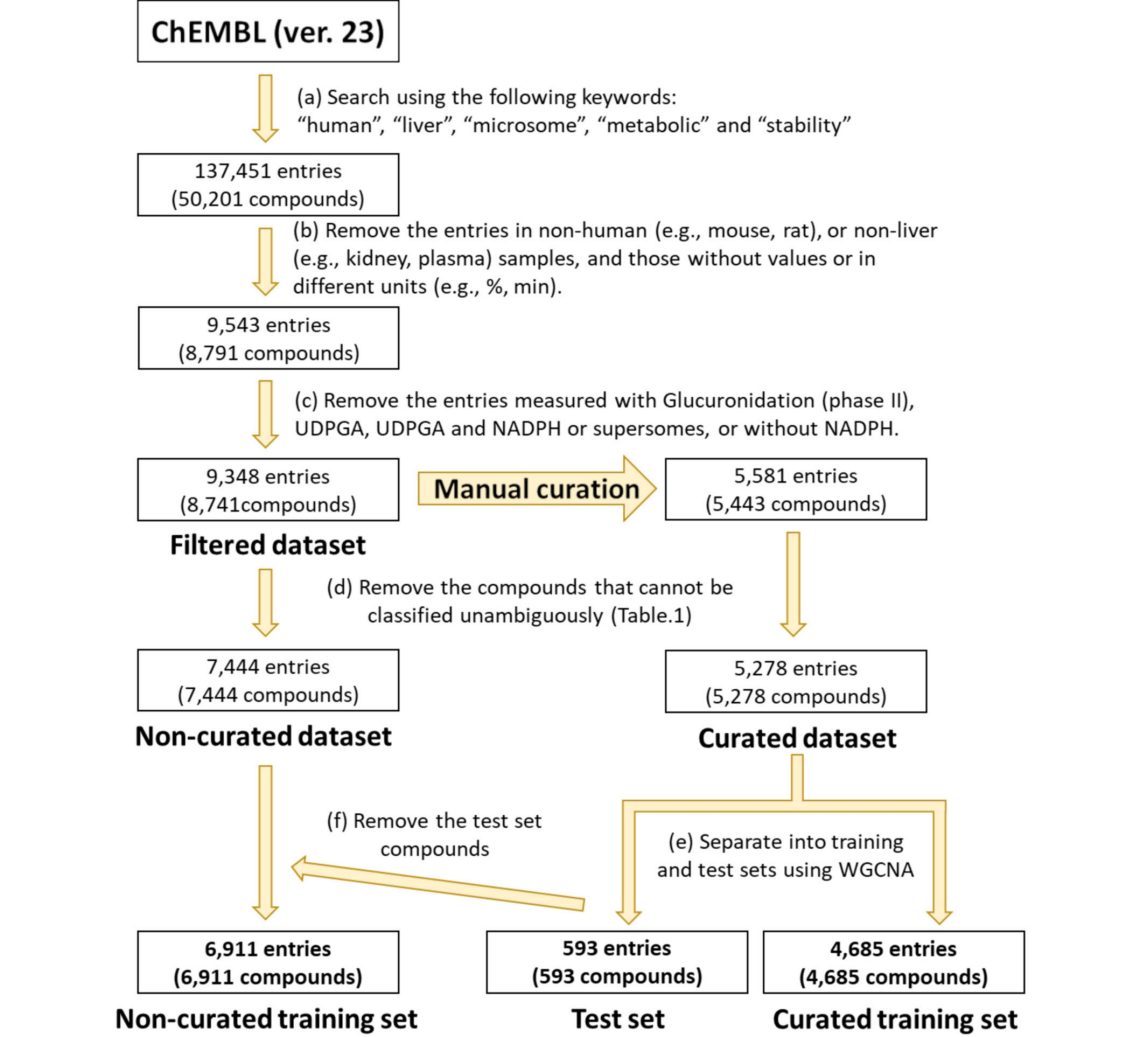
Procedure used for filtering the data to construct two training sets and a test set. To evaluate the efficacy of manual curation, the non‐curated dataset and the curated dataset were constructed. The test set was prepared to evaluate the performance of the models trained on the non‐curated and curated training sets.

A search against the ChEMBL database (ver. 23)^9^ with the keywords shown in Scheme 1 produced 137,451 hits (for 50,201 compounds). We scanned the descriptions and removed the entries that lacked experimental values, were measured in samples different from HLMs, or had non‐*CL*_int_ units (such as % and min), resulting in 9,543 entries (8,791 compounds) remaining. We then checked the experimental protocols and retained only those data that were measured at 37 degrees and pH 7.4, in the absence of inhibitors, and in the presence of reduced NADPH as a cofactor. The filtered dataset comprised 9,348 entries (8,741 compounds). The unit of the collected data was converted to μL/min/mg using the following equation, as proposed by Obach et.al.,^10^ *CL*_int_=(mL incubation)/(mg microsomes)×(45 mg microsomes)/(g liver)×(21 g liver)/(kg body weight) (defined as Equation (1)).

Based on previous studies, we classified the compounds into three categories: stable if *CL*_int_<20 μL/min/mg, moderate if 20≤*CL*_int_<300 μL/min/mg, and unstable if *CL*_int_≥300 μL/min/mg. In this filtered dataset, compounds with multiple entries (reports) belonging to more than one category were removed (Supporting Information). The final “non‐curated dataset” consisted of 7,444 compounds.

### 2.2 Collection of Manually Curated Data

Starting from the filtered dataset above, we performed extensive manual curation (see Results and Discussion) and defined the “curated dataset” consisting of 5,278 compounds.

### 2.3 Descriptors for Predictive Models

We used Mordred^11^ to calculate 1,612 2D descriptors for the collected compounds. Compound‐specific properties, such as physicochemical, molecular modeling, and structural properties in both the non‐curated and the curated datasets were obtained. To add substructure information, extended connectivity fingerprints (ECFP) were calculated using jCompoundMapper.^12^, ^13^ In this study, ECFP_4, containing information about all atoms within a diameter of four chemical bonds, was obtained as 4,096 binary descriptors using the following parameters: Fingerprint algorithm=ECFPVariant, and Distance cut‐off=6. Overall, 5,708 descriptors were calculated for each compound. After removing 816 features with missing values or zero variance, 4,892 features (797 from Mordred and 4,095 from jCompoundMapper) were used in our analysis.

### 2.4 Separation of the Dataset into Training and Test Sets using WGCNA

For model construction and evaluation, the curated dataset was divided into two groups: training and test sets. To evaluate the predictive ability of the models for new compounds, we selected test compounds with low similarity to those in the training set in the following manner.

We used Weighted Gene Co‐expression Network Analysis (WGCNA),^14^ following the work of Prathipati et al., who applied WGCNA for the first time to cheminformatics datasets.^15^ The “WGCNA” package in R (https://www.r‐project.org/) was used with the parameters (threshold=0.9, corType=“bicor,” networkType=“sigmoid,” power=25, minModulesize=3, and deepSplit=4).

**Figure 1:**
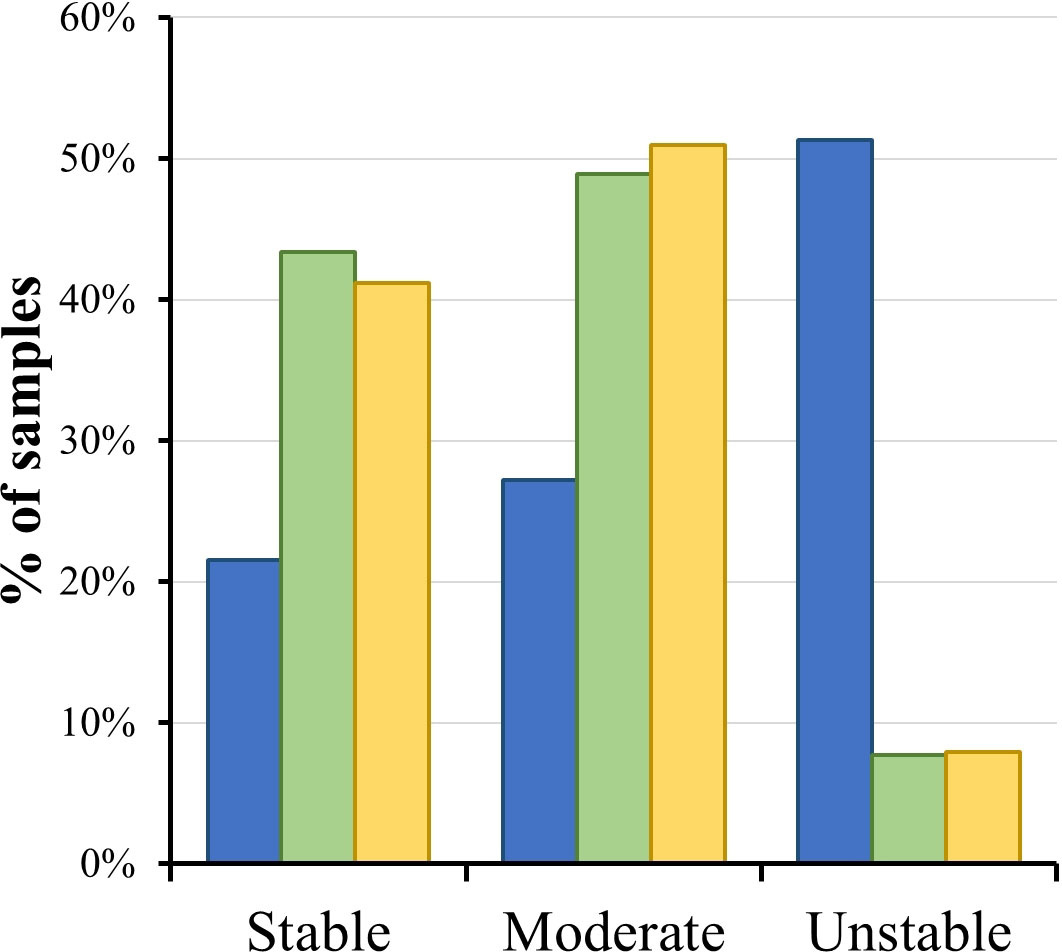
Distribution of the data collected in this study. The lower cut‐off value to define the stable class was chosen to be 20 μL/min/mg, and the upper cut‐off value to define the unstable class was chosen to be 300 μL/min/mg. The bars in blue, green, and yellow represent the non‐curated training set, the curated training set, and the test set, respectively.

The WGCNA clustering of the curated dataset produced three clusters. The largest cluster, including 4,685 compounds (88.8 %), was defined as the “curated training set”. The remaining 593 compounds (11.2 %) in the two smaller clusters were combined and used as the “test set” (Scheme 1). From the non‐curated dataset, the compounds in the test set were removed. The remaining 6,911 compounds were defined as the “non‐curated training set”. Figure 1 shows that all these datasets have similar class distributions.

### 2.5 Selection of Descriptors

To select appropriate features, the “Boruta” package^16^ was used in R. 953 and 725 descriptors were selected from the non‐curated training set and curated training set, respectively, as the important descriptors for classifying the compounds.

### 2.6 Diversity of the Collected Compounds

To determine the diversity of the compounds in our datasets, their chemical profiles were compared with those of the approved drugs. A total of 5,864 approved drugs were obtained from KEGG DRUG^17^ with the filters of “molecular weight≤1,000” and “no inner salt”, as a representative set of drug‐like compounds. These approved drugs, as well as the compounds in our datasets, were mapped in the descriptor space created by 797 features calculated using Mordred, and the principal components analysis (PCA) was performed using the “prcomp” function in R. Furthermore, four properties in Lipinski's rule of five^18^ (molecular weight (MW): <500, logP: <5, hydrogen bond acceptor count (HBA): <10, and hydrogen bond donor count (HBD): <5) were used to evaluate the diversity of these datasets.

### 2.7 Machine Learning Methods

To construct the predictive models, Random Forest (RF), AdaBoost (AB), Support Vector Machine with the radial kernel (Radial SVM) and the linear kernel (Linear SVM), were used in the “caret” package^19^ in R. The general training parameters were as follows: tuneLength=10, validation method=“oob” (out‐of‐bag, for RF) or “10‐fold cv” (10‐fold cross‐validation, for AB, Radial SVM and Linear SVM), and “centering and scaling.”

RF is a machine learning method based on an ensemble of decision trees.^20^ In RF classification, bootstrap samples randomly drawn from the training set are used for training. The retained samples (out‐of‐bag) are used for evaluation. A training parameter, the number of different descriptors tried at each decision point (mtry), was optimized and the best values of mtry were 242 for the non‐curated training set and 101 for the curated training set.

AB is an ensemble classification method based on the boosting algorithm.^21^ The parameters to be optimized were as follows: the number of iterations for which boosting is run or the number of trees to use (mfinal), the depth of the max tree (maxdepth), and the weight updating coefficient type (coeflearn) selected from Breiman, Freund, or Zhu. The values obtained after the optimization were: mfinal=450, maxdepth=10, and coeflearn=Freund for the non‐curated training set and mfinal=500, maxdepth=9, and coeflearn=Breiman.

SVM is a method based on statistical learning theory.^22^ In this study, both the radial kernel and the linear kernel were used. For Radial SVM, two parameters were needed to be optimized: in‐sensitive loss function (sigma) and the cost of constraints violation (cost). The cost parameter was needed to be optimized for Linear SVM. After the optimization of Radial SVM, sigma=0.0006384086 and cost=4 were obtained for the non‐curated training set. This Radial SVM model was trained on 4,529 support vectors. The values of sigma=0.0009252766 and cost=16 were obtained based on 3,036 support vectors in the curated training set. For Linear SVM, cost=1 was used as the optimized parameters for both the non‐curated and curated training sets. The numbers of support vectors used for training were 3,791 for the non‐curated dataset and 2,736 for the curated dataset.

### 2.8 Performance Evaluation

To compare the performance between different models, the prediction results were represented as a confusion matrix and statistical parameters were calculated.^23^ A value of kappa (true accuracy as the agreement by chance is corrected) greater than 0.4 is considered to be indicative of a model with useful predictive power.^3^ Each score defined by the equations in Supporting Information was calculated using the “train” or “confusionMatrix” functions in “caret.”

## 3 Results and Discussion

### 3.1 Construction of the Training and Test Sets

By examining previous studies,^2^,^3^ we defined three classes for the metabolic stability of a compound: “stable (*CL*_int_<20 μL/min/mg),” “moderate (20 μL/min/mg≤*CL*_int_<300 μL/min/mg),” and “unstable (*CL*_int_≥300 μL/min/mg)”. The rationale behind this definition is the following.

If we assume molecules to be in equilibrium between the plasma and blood cells and no binding to liver microsomes, the hepatic clearance (*CL*_h_) and the hepatic availability (*F*_h_) of a drug are shown as: *CL*_h_=(*Q*_h_×*f*_up_×*CL*_int_)/(*Q*_h_+*f*_up_×*CL*_int_) (Equation (2)) and *F*_h_=*Q*_h_/(*Q*_h_+*f*_up_×*CL*_int_) (Equation (3)), where *Q*_h_ is the blood flow in the liver, and *f*_up_ is the fraction of free drug in the plasma.

**Figure 2:**
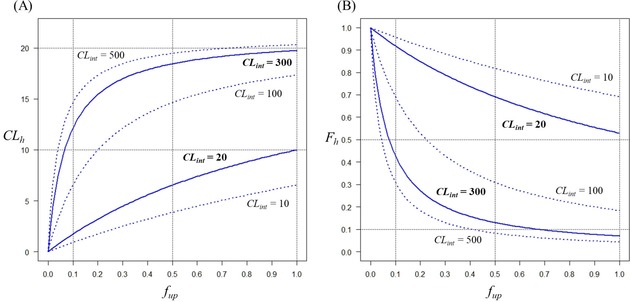
(A) The relationships between *f*_up_ and *CL*_h_. The solid lines represent those with *CL*_int_=20 and 300 μL/min/mg, respectively. The broken lines represent those with *CL*_int_=10, 100 and 500 μL/min/mg, respectively. (B) The relationships between *f*_up_ and *F*_h_. The solid lines represent those with *CL*_int_=20 and 300 μL/min/mg, and the broken lines with *CL*_int_=10, 100 and 500 μL/min/mg, respectively.

The impacts of *CL*_int_ on both *CL*_h_ and *F*_h_ are shown in Figure 2. *CL*_h_ and *F*_h_ were calculated using Equations 2 and 3 by setting *Q*_h_=21.215 mL/min/kg (based on the values of 5.2 L/min for cardiac output, 26.0 % for percent cardiac output of blood flow distribution in the liver, and 64 kg for the average body weight, all in humans).^24^ If a compound has *CL*_int_=20 μL/min/mg, *F*_h_>0.9 with *f*_up_=0.1, and even with *f*_up_=1.0, *F*_h_ is still greater than 0.5. In other words, more than half of the drug reaches the systemic circulation and therefore, it would be reasonable to call such a drug metabolically stable. On the other hand, a compound with *CL*_int_=300 μL/min/mg would have the *F*_h_ values of 0.428, 0.130, and 0.070 for *f*_up_=0.1, 0.5, and 1.0, respectively. This compound can be judged to be unstable and thus, unsuitable as a drug candidate.

We constructed the datasets as described in Material and Methods. Some compounds were associated with two or more measurements belonging to different classes or had a range of values such as “*CL*_int_>20 μL/min/mg”. Since these compounds were not classified unambiguously, 1,297 compounds (1,347 entries) were removed from the filtered dataset. Each of the remaining 7,444 compounds was associated with one class only (Supporting Information) and this collection was defined as a “non‐curated dataset”.

To improve the quality of the dataset, we performed extensive manual curation as follows. First, the experimental protocols were checked and any measurements obtained with protocols different from that we decided to focus on in this study were excluded from the filtered dataset (3,767 entries for 3,298 compounds removed; see Material and Methods for more details). Second, the values and units obtained from the ChEMBL database were cross‐checked with those in the original publications. After visual inspection, we noted that some values were likely to be recorded erroneously in ChEMBL and they were replaced with those reported in the original publications (405 entries for 392 compounds corrected). When ChEMBL used a unit different from that in the original publications, almost all of these entries were associated with incorrectly converted values. These values were recalculated by using equation (1) (3,821 entries for 3,708 compounds corrected). From the remaining 5,581 entries (for 5,443 compounds), 147 compounds were removed, due to the lack of unambiguous class assignments as above. As in the non‐curated dataset, each of the remaining 5,278 compounds was assigned to a single class; this collection was defined as the “curated dataset.”

The curated dataset was clustered using WGCNA, resulting in three clusters (see Material and Methods for details). We defined the largest cluster as the “curated training set” (4,685 compounds; 88.8 %). The remaining 593 compounds (11.2 %) in the two smaller clusters comprised the “test set” (Scheme 1). From the non‐curated dataset, the compounds in the test set were removed, and the remaining 6,911 compounds were defined as the “non‐curated training set”. As shown in Figure 1, the non‐curated training set included considerably more unstable compounds than the curated dataset. This difference was largely due to erroneous records in ChEMBL; for example, the entry ChEMBL1924026 is associated with a value of 42 mL/min/g, which was automatically converted (by using equation 1) to 933.3 μL/min/mg in the non‐curated training set and was labelled unstable. However, the correct value, as in the original publication, was 42  μL/min/mg,^25^ and this compound was classified as moderate in the curated dataset. We made similar corrections for 2,256 entries. This observation suggests that without the manual curation, we would have overestimated the *CL*_int_ values for many entries in ChEMBL.

### 3.2 Distribution of the Collected Dataset

**Figure 3:**
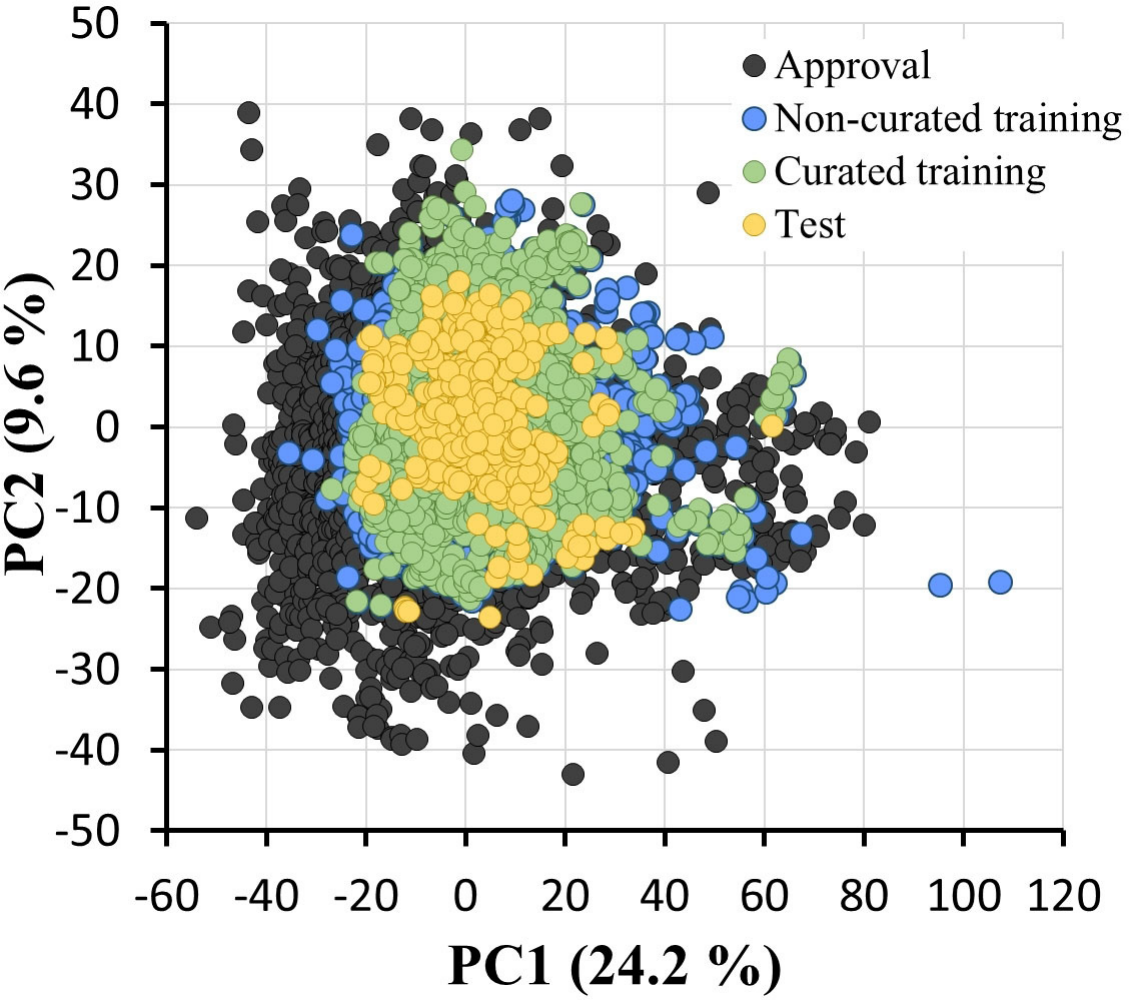
Principal component analysis (PCA) of 797 physicochemical descriptors, representing the chemical space covered by the compounds in the non‐curated training set (6,911 compounds; blue), curated training set (4,685 compounds; green), and test set (593 compounds; yellow), along with a representative set of approved drugs from KEGG DRUG (5,864 compounds; black).

**Figure 4:**
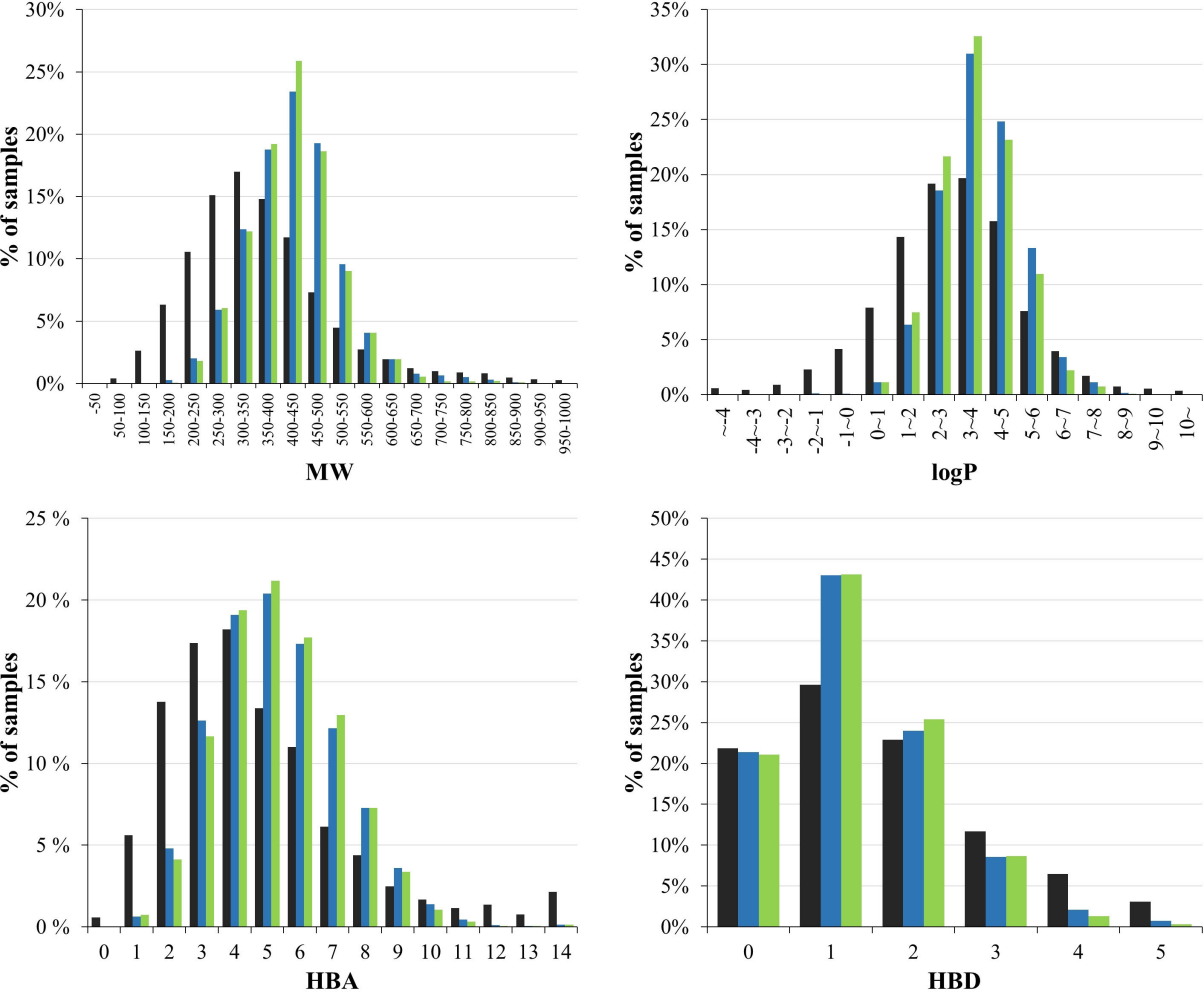
Distribution of physicochemical properties of the collected data compared with a representative set of approved drugs in terms of Lipinski's rule of five. The properties, calculated using Mordred, include molecular weight (MW), calculated log*P* (log*P*), the number of hydrogen bond acceptors (HBA), and the number of hydrogen bond donors (HBD). The bars in black, blue, and green represent approved drugs, non‐curated training set, and curated training set, respectively.

Figure 3 shows a two‐dimensional PCA plot of the approved drug set, non‐curated training set, curated training set, and test set for 797 physicochemical descriptors. The first two principal components explain 33.8 % of variance. The compounds in all of our datasets showed distributions broadly similar to that of the approved drugs. Figure 4 shows that the distributions of physicochemical properties (as used in Lipinski's rule of five) for our datasets are similar to those for the approved drugs. These observations suggest that our training and test sets are appropriate for building and evaluating prediction models for drug‐like compounds.

### 3.3 Classification Performance with Cross‐Validation

We cross‐validated the prediction models trained using the selected set of 953 descriptors on the 6,911 compounds in the non‐curated training set (see Material and Methods). The Radial SVM model produced the highest performance, followed by AB and RF (Table 1).

**Table 1:** Performance summary of the models trained on the non‐curated and curated training sets using different machine learning methods.

	Training set	Method	Accuracy	Kappa
		RF	0.781	0.631
		AB	0.754	0.577
	Non‐curated	Radial SVM	0.780	0.634
		Linear SVM	0.711	0.523
Cross validation		RF	0.757	0.560
		AB	0.766	0.574
	Curated	Radial SVM	0.764	0.576
		Liner SVM	0.715	0.487
		RF	0.300	0.114
		AB	0.317	0.106
	Non‐curated	Radial SVM	0.331	0.103
		Linear SVM	0.3575	0.113
Test set		RF	0.723	0.492
		AB	0.729	0.498
	Curated	Radial SVM	0.771	0.588
		Linear SVM	0.697	0.455

We also evaluated the prediction models trained with 725 descriptors on the 4,685 compounds in the curated training set. The AB and Radial SVM models showed the highest performance for accuracy and kappa, respectively.

Using the same learning algorithm, the model trained on the non‐curated training set performed better than that trained on the curated training set in all the cases. The Linear SVM model showed the lowest predictability with both the non‐curated and curated training sets (Table 1). As Table 2 shows, descriptors that had a high contribution to the RF prediction included physicochemical properties such as SLogP and TopoPSA. The metabolic stability of compounds depends on complex factors involving their physicochemical properties and interactions with enzymes such as cytochrome P450 isoforms. It appears that ensemble or nonlinear learning algorithms (RF, AB and Radial SVM) capture these factors better than the linear method (Linear SVM) in the current analysis.

**Table 2:** A list of the most important descriptors with the ten highest feature importance scores are shown out of the 953 (non‐curated training set) and 725 (curated training set) descriptors from the RF models.

Symbol	Description	Scaled importance in non‐curated model (%)^a^	Scaled importance in curated model (%)^a^
SLogP	Wildman‐Crippen LogP	100	100
NsssCH	Number of sssCH	83.91	0.744^b^
JGI8	8‐orderd mean topological charge	77.51	6.60^b^
JGI4	4‐orderd mean topological charge	74.27	6.63 ^b^
SdssC	Sum of dssC in Atom Type of EState	66.88	27.47
ATS8v	Moreau‐broto autocorrelation of lag 7 weighted by vdw volume	65.97	19.85
TopoPSA (NO)	Topological polar surface area (use only nitrogen and oxygen)	52.62	27.07
Xch‐7dv	7‐orderd Chi chain weighted by valence electrons	49.54	6.46^b^
PEOE_VSA6	MOE charge VSA Descriptor 9	49.51	18.36
nAcid	Acidic group count	48.02	17.51
AATSC3pe	Averaged and centered moreau‐broto autocorrelation of lag 3 weighted by sanderson EN	20.57^b^	19.06
AATSC3se	Averaged and centered moreau‐broto autocorrelation of lag 3 weighted by sanderson EN	27.98^b^	18.84
Sd0	Sum of dO	39.67^b^	18.54
TopoPSA	Topological polar surface area	31.07^b^	16.84

^a^ The scaled importance of the descriptors was calculated using the RF model. ^b^ Indicating a descriptor outside the top 10 list.

### 3.4 External Validation with Test Set

**Figure 5:**
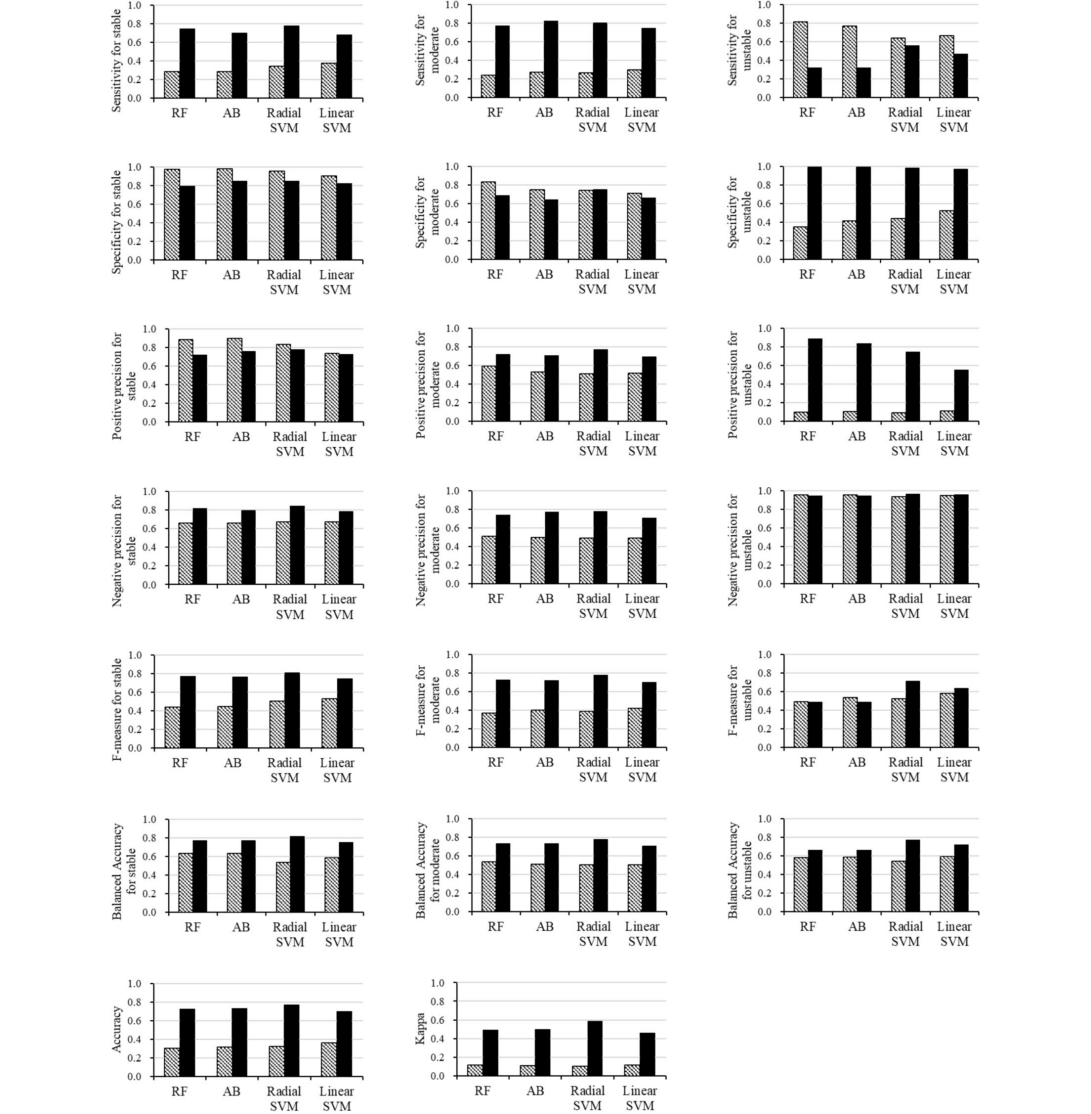
The complete set of performance metrics on the test set for the models trained on different datasets and using different machine learning methods. The following learning algorithms were used: RF, AB, Radial SVM and Linear SVM. The hatched and solid bars represent the results of the non‐curated and the curated training sets, respectively.

External validation on the test set was performed for each model. Table 1 shows the accuracy and kappa values calculated on the test set and compared them with the cross‐validation results described above. The complete set of statistical scores are shown in Figure 5.

Irrespective of the learning algorithm used, the models trained on the non‐curated training set tended to overpredict the unstable class (Table 3). On the other hand, the classifiers trained on the curated training set more correctly classified the test data in each category. The Radial SVM model trained on the curated training set produced the highest scores in terms of both accuracy and kappa for the test set. The scores of accuracy and kappa in the external validation were similar to those in the cross‐validation. The RF, AB and Radial SVM models showed high accuracy and kappa compared to the Linear SVM models, confirming the observations described above.

**Table 3:** Confusion matrix of each model (trained on the non‐curated training set (the upper table) and on the curated training set (the lower table)) evaluated using the test compounds.

Non‐ curated	Obs.	Pred. Stable	Pred. Moderate	Pred. Unstable	Critical mis‐predictio n (%)^a^
RF	Stable	69	41	134	22.77
	Moderate	8	71	223	
	Unstable	1	8	38	
AB	Stable	70	64	110	18.72
	Moderate	7	82	213	
	Unstable	1	10	36	
Radial SVM	Stable	83	60	101	17.20
	Moderate	16	79	207	
	Unstable	1	16	30	
Linear SVM	Stable	91	73	80	14.17
	Moderate	29	90	183	
	Unstable	4	12	31	
RF	Stable	181	63	0	0.51
	Moderate	67	233	2	
	Unstable	3	29	15	
AB	Stable	170	74	0	0.34
	Moderate	52	247	3	
	Unstable	2	30	15	
Radial SVM	Stable	190	54	0	0.34
	Moderate	52	241	9	
	Unstable	2	19	26	
Linear SVM	Stable	166	76	2	0.51
	Moderate	61	225	16	
	Unstable	1	24	22	

^a^ The rightmost column in these tables shows the percentage of critical mis‐prediction, defined as: (the number of unstable compounds predicted to be stable)+(the number of stable compounds predicted to be unstable)/(the total number of the test compounds).

We defined the critical mis‐prediction as: (the number of unstable compounds predicted to be stable)+(the number of stable compounds predicted to be unstable)/(the total number of the test compounds) (Table 3). The critical mis‐prediction was lower on the curated data; all the models trained on the non‐curated training set predicted many of the stable compounds as unstable, likely due to the erroneous records in ChEMBL.

Overall, the models on the curated training set had higher prediction capabilities than those on the non‐curated training set against the test set (Table 1, Figure 5). Even after removing the effects of unequal class distributions between the curated and non‐curated training sets, the models trained on the curated training set still produced considerably better prediction results (Supporting Information). To our knowledge, this result is the first explicit demonstration of how manual curation can improve the performance of prediction models. Presumably, the manual curation reduced the noise, for example, in the forms of experimental measurements with different protocols, incorrect unit conversion, and erroneous recordings, and contributed to more effective learning.

### 3.5 Comparison with Other Classification Models

It is difficult to directly compare our models with other models from the literature, because neither the program nor the detailed training set are available in most cases. In general, the performance of the models depends on the number, diversity, and composition of the training data compounds. Furthermore, the published models adopted different cut‐off values for classification, because they were developed for different purposes. Thus, we simply quote accuracy and kappa for the test set to place our models in the context of published studies (Table 4).

**Table 4:** Comparison with previous studies.

Models^a^	Cut‐off Values	Number of Training set	Accuracyc^b^	Kappa^b^
P. H. Lee [2]	20 μL/ min/mg	11,646 (In‐house data)	0.82	0.64
Y. Sakiyama [3]	20 mL/ min/kg (21.164 μL/min/mg)	1,952 (In‐house data)	0.72	0.71
R.R. Gupta [4]	13 μL/ min/mg 50 μL/ min/mg	49,968 (In‐house data)	(no data)	0.43
Curated Radial SVM (This study)	20 μL/ min/mg 300 μL/ min/mg	4,685 (curated data from ChEMBL)	0.771	0.588

^a^ The two upper models are two‐classification models, other two lower are three‐classification models. ^b^ The accuracy and kappa of previous studies were taken from the literature, and those from our models were the test set results.

In previous studies, Lee et al. proposed an RF classifier to classify in‐house compounds into two categories using ADME keys,^2^ with the best performance of accuracy=0.82 and kappa=0.64. Sakiyama et al. also used in‐house library compounds to classify test compounds into two groups with Molecular Operating Environment for descriptors calculation.^3^ The RF yielded the highest scores: accuracy=0.72 and kappa=0.71. A three‐class model was developed by Gupta et al,^4^ and the compounds collected from an in‐house project were used to train and evaluate it (kappa=0.43) by using Chemistry Developers Kit. These previous studies indicate that accuracy >0.72 and kappa >0.43 constitute acceptable performance for metabolic stability prediction. Our Radial SVM model on the curated training set had accuracy=0.771 and kappa=0.588 and thus satisfied both the accuracy and kappa criteria.

## 4 Conclusions

In this paper, we proposed metabolic stability prediction models for classifying compounds into three categories based on *CL*_int_. In all our analysis, the *CL*_int_ data used were collected from an open data source. By curating the collected data carefully, we were able to build effective classifiers that are on par with or had better performance than previously published models. The curated data have been made available as supporting information to help reproduce our study widely.

This is the first study to investigate the effect of manual curation of data obtained from an open data source. This result can open the possibility for broad research communities to access high‐quality pharmacokinetic datasets and build their own models.

## Supplementary Materials

1) Supporting Information_Details_Esaki et al.pdf: The PDF file involves the details of removing compounds because of the unambiguous class assignments (S‐2). The confusion matrix and equations for calculating each statistical score (S‐3) and the results of models trained on the sampled non‐curated data set (S‐4 and S‐5) also are included.

2) Supporting Information_Structural data_Esaki et al.sdf: Structure data file of the curated data set includes the information of ChEMBL ID, Compound name, Dataset (training or test sets), Observed and Predicted classes with Radial SVM model trained on the curated training set.

## Conflict of Interest

None declared.

## Supporting information

As a service to our authors and readers, this journal provides supporting information supplied by the authors. Such materials are peer reviewed and may be re‐organized for online delivery, but are not copy‐edited or typeset. Technical support issues arising from supporting information (other than missing files) should be addressed to the authors.

Supplementary

Supplementary
