# Supplementary material for: Data Curation can Improve the Prediction Accuracy of Metabolic Intrinsic Clearance
Source: Mol Inform. 2018 Sep 24;38(1-2):1800086. doi: 10.1002/minf.201800086 (PMC6586005; doi:10.1002/minf.201800086)
Supplement: Supplementary file 1 — Supplementary [file MINF-38-na-s001.pdf]

Supporting Information

Data curation can improve the prediction accuracy of metabolic intrinsic clearance

Tsuyoshi Esaki,*[a] Reiko Watanabe, [a] Hitoshi Kawashima, [a] Rikiya Ohashi, [a], [b] Yayoi Natsume-Kitatani, [a], [c] Chioko Nagao, [a], [c] Kenji Mizuguchi, *[a], [c]

[a] Laboratory of Bioinformatics, National Institutes of Biomedical Innovation, Health and Nutrition, 7-6-8 Saito-Asagi, Ibaraki, Osaka 567-0085, Japan

[b] Discovery Technology Laboratories, Mitsubishi Tanabe Pharma Corporation, 2-2-50 Kawagishi, Toda, Saitama 335-8505, Japan

[c] Laboratory of In-silico Drug Design, Center for Drug Design Research, National Institutes of Biomedical Innovation, Health and Nutrition, 7-6-8 Saito-Asagi, Ibaraki, Osaka 567-0058, Japan

S-1. Title page

S-2. Automated initial processing.

S-3. Confusion matrix and equations for performance metrics.

S-4. Performance of models trained on the sampled non-curated data set.

S-5. Detailed prediction results of models trained on the sampled non-curated data set.

Automated initial processing

Relation	Value<br>(μL/min/mg)	Category	Number of entries in<br>Non-curated dataset	Number of entries in<br>Curated dataset
< or ≤	0–20	Stable	457	901
=	0–20	Stable	1,318	1,496
>	0–20	Removed	16	9
< or ≤	20–300	Removed	1,074	77
=	20–300	Moderate	2,174	2,621
>	20–300	Removed	24	59
<	300~	Removed	233	9
=	300~	Unstable	3,685	302
>	300~	Unstable	367	107
Removed entries			1,347	154
Retained entries			8,001	5,427
Total			9,348<br>(8,741 compounds)	5,581<br>(5,443 compounds)

Automated initial processing of the entries in the non-curated and curated datasets. The grayed cells indicate the entries that have been removed. The sums of the white cells represent the number of entries in each category in each dataset.

Confusion matrix and equations for performance metrics

		Predicted		
		Stable	Moderate	Unstable
Observed	Stable	True Stable **A**	False Moderate **B**	False Unstable **C**
	Moderate	False Stable **D**	True Moderate **E**	False Unstable **F**
	Unstable	False Stable **G**	False Moderate **H**	True Unstable **I**

**Measure**	**Calculation**
Specificity	Stable $\frac{E+F+H+I}{D+E+F+G+H+I}$, Moderate $\frac{A+C+G+I}{A+B+C+G+H+I}$, Unstable $\frac{A+B+D+E}{A+B+C+D+E+F}$
Positive Predicted Value	Stable $\frac{A}{A+D+G}$, Moderate $\frac{E}{B+E+H}$, Unstable $\frac{I}{C+F+I}$
Negative Predicted Value	Stable $\frac{E+F+H+I}{B+C+E+F+H+I}$, Moderate $\frac{D+G+F+I}{A+D+G+C+F+I}$, Unstable $\frac{A+B+D+E}{A+D+G+B+E+H}$
Sensitivity (Recall)	Stable $\frac{A}{A+B+C}$, Moderate $\frac{E}{D+E+F}$, Unstable $\frac{I}{G+H+I}$
F-measure (for class *i*)	$\frac{2(\text{Sensitivity}_i \times \text{Precision}_i)}{\text{Sensitivity}_i + \text{Precision}_i}$
Balanced Accuracy	Stable $\frac{1}{2}\left(\frac{A}{A+B+C}+\frac{E+F+H+I}{D+E+F+G+H+I}\right)$,<br>Moderate $\frac{1}{2}\left(\frac{E}{D+E+F}+\frac{A+C+G+I}{A+B+C+G+H+I}\right)$,<br>Unstable $\frac{1}{2}\left(\frac{I}{G+H+I}+\frac{A+B+D+E}{A+B+C+D+E+F}\right)$
Accuracy	$\frac{A+E+I}{A+B+C+D+E+F+G+H+I}$
Kappa	$\frac{\text{Accuracy}-E}{1-E}$
E	$\frac{(A+B+C)(A+D+G)+(D+E+F)(B+E+H)+(G+H+I)(C+F+I)}{(A+B+C+D+E+F+G+H+I)^2}$

The upper table explains a confusion matrix, where A to I represent the number of instances. The lower table shows equations for the performance metrics, calculated based on the confusion matrix[1].

---

[1] Sokolova, M.; Lapalme, G. A systematic analysis of performance measures for classification tasks. *Inf. Process. Manag.* **2009**, *45*, 427-437.

Performance of models trained on the sampled non-curated data set.

	Training data	Method	Accuracy	Kappa
Cross validation	Reconstructed non-curated data	RF	0.744	0.526
		AB	0.740	0.517
		Radial SVM	0.754	0.556
		Linear SVM	0.687	0.453
Test set	Reconstructed non-curated data	RF	0.573	0.192
		AB	0.563	0.174
		Radial SVM	0.548	0.216
		Linear SVM	0.528	0.153

A sampled non-curated dataset (stable: 1,486 compounds (43.39%), moderate: 1,674 compounds (48.88%), unstable: 265 compounds (7.73%)) was prepared by randomly sampling the non-curated training set to make its class distribution nearly identical to that of the curated training set. The cross-validation results were similar to those for the models trained on the original (unsampled) non-curated training set. While the performance scores on the test set improved, they were still worse than those produced by the models trained on the curated data (Table 1).

Detailed prediction results of models trained on the sampled non-curated data set.

Reconstructed non-curated			Predicted			Critical mis-prediction (%)
			Stable	Moderate	Unstable	
RF	Observed	Stable	103	138	3	1.18
		Moderate	69	231	2	
		Unstable	4	37	6	
AB	Observed	Stable	106	138	0	0.51
		Moderate	78	221	3	
		Unstable	2	38	7	
Radial SVM	Observed	Stable	119	114	11	2.87
		Moderate	69	188	45	
		Unstable	6	23	18	
Linear SVM	Observed	Stable	107	130	7	2.19
		Moderate	79	194	29	
		Unstable	6	29	12	

The confusion matrix shows the prediction results of the models trained on the sampled non-curated dataset. The AB model showed an improved mis-prediction ratio but RF, Radial SVM and Linear SVM model produced worse results (compared with Table 3).
